# In vitro activity of biofilm inhibitors in combination with antibacterial drugs against extensively drug-resistant *Acinetobacter baumannii*

**DOI:** 10.1038/s41598-020-75218-y

**Published:** 2020-10-22

**Authors:** Qin Peng, Fei Lin, Baodong Ling

**Affiliations:** 1 Sichuan Province College Key Laboratory of Structure-Specific Small Molecule Drugs, Chengdu Medical College, Chengdu, 610500 China; 2 School of Pharmacy, Chengdu Medical College, Chengdu, 610500 China; 3 Clinical Medical College and The First Affiliated Hospital of Chengdu Medical College, Chengdu, 610500 China; 4 Present Address: Department of Pharmacy, Nanchong Central Hospital, Nanchong, 637000 China

**Keywords:** Drug discovery, Microbiology

## Abstract

*Acinetobacter baumannii* is a common pathogen of nosocomial infection, and its ability to form biofilms further contributes to its virulence and multidrug resistance, posing a great threat to global public health. In this study, we investigated the inhibitory effects of five biofilm inhibitors (BFIs) (zinc lactate, stannous fluoride, furanone, azithromycin, and rifampicin) on biofilm formation of nine extensively drug-resistant *A. baumannii* (XDRAB), and assessed the synergistic antibacterial effects of these BFIs when combined with one of four conventional anti-*A. baumannii* antibiotics (imipenem, meropenem, tigecycline, and polymyxin B). Each of the five BFIs tested was found to be able to significantly inhibit biofilm formation of all the clinical isolates tested under sub-minimal inhibitory concentrations. Then, we observed synergistic effects (in 22%, 56% and 11% of the isolates) and additive effects (56%, 44% and 44%) when zinc lactate, stannous fluoride and furanone were combined with tigecycline, respectively. When zinc lactate and stannous fluoride were each used with a carbapenem (imipenem or meropenem), in 33% and 56–67% of the isolates, they showed synergistic and additive effects, respectively. Additivity in > 50% of the isolates was detected when rifampicin was combined with imipenem, meropenem, tigecycline, or polymyxin B; and a 100% additivity was noted with azithromycin-polymyxin B combination. However, antagonism and indifference were noted for polymyxin B in its combination with zinc lactate and stannous fluoride, respectively. In conclusion, five BFIs in combination with four antibacterial drugs showed different degrees of in vitro synergistic and additive antibacterial effects against XDRAB.

## Introduction

*Acinetobacter baumannii* is a major gram-negative opportunistic pathogen causing hospital infections^1^. Clinical isolates of *A. baumannii* not only possess strong intrinsic resistance to a variety of structurally unrelated antibiotics but also show increasing acquired multidrug or extensive drug resistance^1,2^. In particular, carbapenem-resistant *A. baumannii* has been categorized by the World Health Organization as one of the 12 top priority resistant bacteria that pose the greatest threat to human health with the urgent need for developing new antibiotics^3^. *A. baumannii* also possesses strong biofilm formation ability, which further enhances its resistance to antibacterial drugs and circumvents the host immune-mediated clearance, and thus greatly contributes to recurrent infections or chronic infections^4–7^.

Biofilms are surface-attached population of bacterial cells, which are encased in self-produced extracellular polymers. The latter mainly includes a range of substances such as polysaccharides, proteins, nucleic acids, and lipids, forming a dense physical barrier that renders, along with other mechanisms, biofilm cells much more resistant to antibacterial drugs than their non-adherent planktonic cells^8,9^. In recent years, much attention has been directed to uncovering new approaches including combination therapy against biofilm infections^10,11^.

Control of bacterial biofilm formation is considered as one major strategy against bacterial infections, and thus efforts have been made to identify effective inhibitors that go beyond conventional antibiotics for their ability to disrupt biofilm formation^10^. For example, it has been reported that three chemical substances, zinc lactate, stannous fluoride and furanone, and two antibiotics, azithromycin and rifampicin, can inhibit bacterial biofilm formation, but cannot effectively control the infection caused by biofilm-forming bacteria^12–17^. Yet, information available is largely not specific to *A. baumannii*. In this study, we aimed to assess inhibitory effects of five aforementioned biofilm inhibitors (BFIs) on the biofilm formation of extensively drug-resistant *A. baumannii* (XDRAB) and to explore if these inhibitors could synergistically interplay with one of four conventional anti-*A. baumannii* antibiotics (imipenem, meropenem, tigecycline or polymyxin B) against XDRAB. The results obtained warrant further investigations towards the combination strategy for their potential in the prevention and treatment of the infections caused by biofilm-forming XDRAB.

## Results

### Antimicrobial susceptibility testing

Antibacterial activity of 15 antibiotics against nine clinical isolates of *A. baumannii* is shown in Table 1. These tested agents include a variety of structurally unrelated antibiotics belonging to nine classes or subclasses of β-lactam-β-lactamase inhibitors, aminoglycosides, carbapenems, cephalosporins, fluoroquinolones, glycylcyclines, phenicols, polymyxins, and tetracyclines. Most of these antibiotics tested are clinically relevant as drugs of choice in the treatment of *A. baumannii* infections^1,18^. According to the interpretive categories established by the Clinical and Laboratory Standards Institute (CLSI)^18^, these isolates were either susceptible or non-susceptible to tigecycline and/or polymyxin B, but were resistant to other 13 antibacterial drugs (Table 1), and such drug resistance profiles placed these isolates as XDRAB^19^. In addition, the five BFIs selected for this study, zinc lactate, stannous fluoride, furanone, azithromycin, and rifampicin, displayed different degrees of antibacterial effects on these XDRAB, with identical MIC values of 512, 512, 256, 1024, and 1 µg/ml, respectively, for all nine isolates tested. Based on these MIC values, except for rifampicin which antibacterial activity is demonstrated, activity of zinc lactate, stannous fluoride, furanone and azithromycin against *A. baumannii* is consider limited.

**Table 1:** Activity of 15 antibiotic drugs against 9 clinical isolates of *A. baumannii.*

Antibiotic	MIC (µg/ml) for isolate									Resistance phenotype^a^ (%)
	AB1	AB2	AB3	AB4	AB5	AB6	AB7	AB8	AB9	
Ampicillin	> 1024	> 1024	> 1024	> 1024	> 1024	> 1024	> 1024	> 1024	> 1024	R (100%)
Ampicillin/sulbactam	64/32	128/64	32/16	128/64	64/32	64/32	64/32	32/16	32/16	R (100%)
Ceftazidime	256	128	128	128	128	256	256	128	128	R (100%)
Cefepime	128	32	32	64	32	64	128	64	32	R (100%)
Doxycycline	64	64	64	64	64	64	64	64	64	R (100%)
Minocycline	16	16	16	16	16	16	16	16	16	R (100%)
Amikacin	> 1024	> 1024	> 1024	> 1024	> 1024	> 1024	> 1024	> 1024	> 1024	R (100%)
Gentamicin	> 1024	> 1024	> 1024	> 1024	> 1024	> 1024	> 1024	> 1024	> 1024	R (100%)
Ciprofloxacin	64	128	128	64	64	128	32	128	512	R (100%)
Levofloxacin	16	16	32	16	16	8	32	8	32	R (100%)
Chloramphenicol	128	128	128	128	128	128	64	128	256	R (100%)
Imipenem	64	64	128	64	64	64	64	64	64	R (100%)
Meropenem	32	64	64	64	64	64	32	32	32	R (100%)
Tigecycline	1	4 (NS)	2	2	2	1	1	2	1	S (89%) or NS (11%)
Polymyxin B	2 (I)	1	1	2 (I)	2 (I)	2 (I)	0.5	2 (I)	2 (I)	S (33%) or I (67%)

^a^The standards from the CLSI^18^ for antimicrobial susceptibility phenotypes are listed below with the MIC values in µg/ml included in the brackets, respectively, for susceptible (S), intermediate (I) and resistant (R) (except for situation where intrinsic resistance exists as defined by CLSI): ampicillin (intrinsic resistance), ampicillin-sulbactam (≤ 8/4, 16/8, ≥ 32/16), cefepime and ceftazidime (≤ 8, 16, ≥ 32), doxycycline and minocycline (≤ 4, 8, ≥ 16), amikacin (≤ 16, 32, ≥ 64), gentamicin (≤ 4, 8, ≥ 16), ciprofloxacin (≤ 1, 2, ≥ 4), levofloxacin (≤ 2, 4, ≥ 8), chloramphenicol (intrinsic resistance), imipenem and meropenem (≤ 2, 4, ≥ 8), polymyxin B (not available for S, ≤ 2, ≥ 4). No CLSI interpretative categories are available for tigecycline and the information from US Food and Drug Administration is used for defining *A. baumannii* as S (MIC of ≥ 4 µg/ml) or non-susceptible (NS) (MIC of ≥ 4 µg/ml)^50^.

### Sub-inhibitory concentrations of the biofilm inhibitors

**Figure 1:**
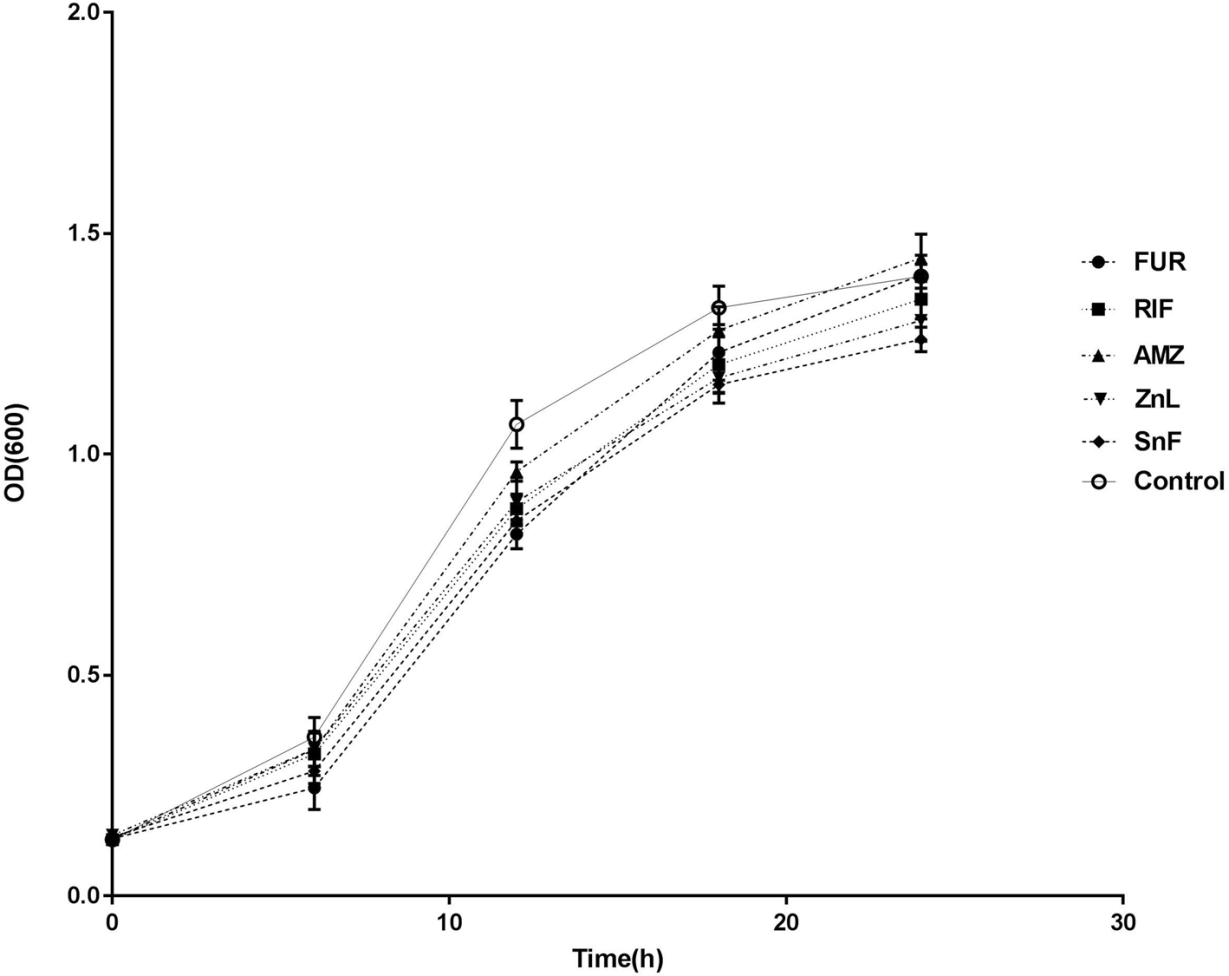
Bacterial growth curves of 9 extensively drug-resistant *A. baumannii* in the presence of sub-inhibitory concentrations of 5 biofilm inhibitors tested. Control, no biofilm inhibitor; *AMZ* azithromycin (1/4 MIC = 256 µg/ml), *FUR* furanone (1/8 MIC = 32 µg/ml), *RIF* rifampicin (1/4 MIC = 0.25 µg/ml), *SnF* stannous fluoride (1/2 MIC = 256 µg/ml), and *ZnL* zinc lactate (1/2 MIC = 256 µg/ml). Data are shown as mean ± SD (n = 9).

Based on literature information^12–17^, we have identified a range of metal salts, biocides and conventionally used antibiotics with ability to affect bacterial biofilm formation. However, no or little data were specifically available to *A. baumannii*. We found that zinc lactate, stannous fluoride, furanone, azithromycin, and rifampicin did not affect the growth of planktonic bacterial cells at the sub-inhibitory concentrations (i.e., 1/8–1/2 MICs) of 256, 256, 32, 256, and 0.25 µg/ml, respectively (Fig. 1). Therefore, these concentrations were established as the working concentrations of the BFIs for assessing their effects on biofilm formation and combination use with anti-*A. baumannii* drugs.

### Effects of sub-inhibitory concentrations of BFIs on XDRAB biofilm formation

**Figure 2:**
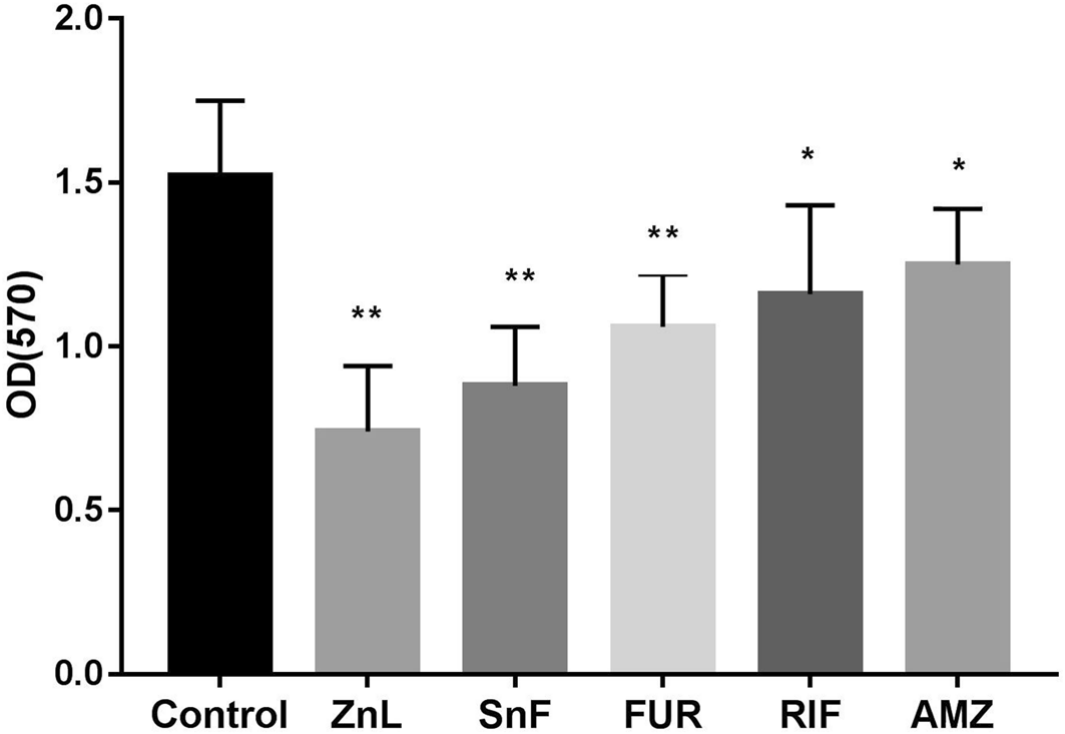
Effects of the sub-inhibitory concentration of 5 biofilm inhibitors on the biofilm formation of 9 isolates of extensively drug-resistant *A. baumannii* as measured via the decolorization solution of crystal violet stained biofilm cells. Control, cells with no biofilm inhibitor; *AMZ* azithromycin (1/4 MIC = 256 µg/ml), *FUR* furanone (1/8 MIC = 32 µg/ml), *RIF *rifampicin (1/4 MIC = 0.25 µg/ml), *SnF* stannous fluoride (1/2 MIC = 256 µg/ml); *ZnL* zinc lactate (1/2 MIC = 256 µg/ml). Data are shown as mean ± SD (n = 9; significance **P < 0.01 and *P < 0.05 calculated by *t*-test).

With the sub-inhibitory concentrations of 5 BFIs described above, we tested the effects of these BFIs on biofilm formation. Compared with the no BFI control group, at the sub-inhibitory concentration of each agent, the five BFIs were found to significantly inhibit the biofilm formation of all 9 XDRAB isolates (*P* < 0.05 or < 0.01) (Fig. 2). The strongest effect was observed with zinc lactate (mean OD_570_ decreased of 0.78, from 1.52 to 0.74), followed by stannous fluoride, furanone, and rifampicin. The weakest effect was observed when azithromycin was used, with an average value of OD_570_ decrease of 0.27 (from 1.52 to 1.25) (Fig. 2). At the assay conditions of this study, the inhibitory effects of the five tested BFIs on the biofilm formation of XDRAB are ranked as: zinc lactate > stannous fluoride > furanone > rifampicin > azithromycin.

### Combination antimicrobial drug susceptibility

Given the observed effects from sub-inhibitory BFIs on biofilm formation, we further tested how a combination use of a BFI with a clinically-relevant anti-*A. baumannii* antibiotic could interplay. Based on the fractional inhibitory concentration index (FICI) values generated from the combination drug susceptibility testing (Table 2), we observed that when zinc lactate was used in combination with imipenem, meropenem, and tigecycline respectively, 33%, 33%, and 22% of the isolates showed synergistic effects, and 67%, 67%, and 56% showed additive effects. However, when combined with polymyxin B, we detected an antagonistic effect. When stannous fluoride was used with imipenem, meropenem, and tigecycline, synergism was detected in 33%, 44%, and 56% of the isolates, respectively, while additivity was noted in 67%, 56%, and 44% of the isolates. Its combination with polymyxin B resulted in indifferent effects. Additivity was detected when furanone was combined with imipenem, meropenem or tigecycline (100%, 100% or 44% respectively). The combination of furanone and polymyxin B yielded either indifferent or antagonistic effect. Combination of rifampicin with imipenem, meropenem, tigecycline or polymyxin B led to additive effects (78%, 56%, 67% and 100%, respectively). When azithromycin was combined with polymyxin B, we observed an additive effect. Yet, azithromycin showed indifferent effects when combined with imipenem, meropenem or tigecycline.

**Table 2:** Antibacterial effect of 5 biofilm inhibitors in combination with anti-*A. baumannii* antibiotics on extensively drug-resistant *A. baumannii.*

Biofilm inhibitor	Antibiotic	Fractional Inhibitory Concentration Index (FICI)									Interplay			
		AB1	AB2	AB3	AB4	AB5	AB6	AB7	AB8	AB9	Synergy (%)	Additivity (%)	Indifference (%)	Antagonism (%)
Zinc lactate +	Imipenem	0.75	0.75	0.75	0.75	0.75	0.38	0.62	0.50	0.50	33	67	0	0
	Meropenem	0.75	1.00	0.75	0.75	0.56	0.75	0.50	0.50	0.38	33	67	0	0
	Tigecycline	0.31	1.06	0.56	0.31	1.06	0.56	0.56	0.56	0.56	22	56	22	0
	Polymyxin B	2.06	4.12	4.12	3.00	4.12	2.06	2.06	2.06	2.06	0	0	0	100
Fluoride +	Imipenem	0.56	0.31	0.56	1.00	1.00	0.56	0.31	0.56	0.31	33	67	0	0
	Meropenem	0.56	0.56	0.56	0.63	0.5	0.56	0.31	0.31	0.31	44	56	0	0
	Tigecycline	0.19	0.56	0.31	0.31	0.56	0.56	0.31	0.56	0.31	56	44	0	0
	Polymyxin B	1.06	1.12	1.12	1.12	1.12	1.25	1.12	1.25	1.12	0	0	100	0
Furanone +	Imipenem	0.56	0.56	0.56	0.56	0.75	0.63	0.63	0.56	0.56	0	100	0	0
	Meropenem	0.56	0.56	0.63	0.56	0.56	0.56	0.56	0.56	0.56	0	100	0	0
	Tigecycline	0.31	1.06	0.56	0.56	1.12	1.06	0.56	1.06	0.56	11	44	44	0
	Polymyxin B	1.25	1.25	2.5	1.25	1.12	4.00	1.25	1.25	1.25	0	0	78	22
Rifampicin +	Imipenem	1.00	1.06	0.53	0.63	0.53	1.06	0.56	0.56	0.56	0	78	22	0
	Meropenem	0.53	0.53	0.53	1.03	1.03	1.06	1.06	0.56	0.53	0	56	44	0
	Tigecycline	0.5	1.00	0.56	0.56	0.56	0.56	1.12	0.50	0.56	22	67	11	0
	Polymyxin B	0.56	0.53	0.53	0.56	0.56	1.03	0.56	0.63	0.56	0	100	0	0
Azithromycin +	Imipenem	1.03	1.03	1.03	1.03	1.03	1.03	1.03	1.03	1.03	0	0	100	0
	Meropenem	1.03	1.03	1.03	1.03	1.03	1.03	1.03	1.03	1.03	0	0	100	0
	Tigecycline	1.03	1.03	1.03	1.03	1.03	1.03	1.03	1.03	1.03	0	0	100	0
	Polymyxin B	0.62	0.62	0.73	0.73	0.73	0.73	0.73	0.73	0.73	0	100	0	0

## Discussion

Our antimicrobial susceptibility testing results show that all nine isolates tested were XDRAB (i.e., non-susceptible to ≥ 1 agent in all tested drug categories but ≤ 2 categories)^19^, with resistance to commonly used antibacterial drugs, including β-lactam plus β-lactamase inhibitor, carbapenems, third/fourth-generation cephalosporins, aminoglycosides, and fluoroquinolones^18^. Some of the isolates were even non-susceptible to tigecycline and/or polymyxin B, which rendered these isolates close to be pandrug-resistant (i.e., non-susceptible to all tested drug categories)^19^. These findings are consistent with the fact that XDRAB has been increasingly isolated in clinical settings globally, which calls for combination therapy^11^. Indeed, the clinical choice of antibacterial drugs for XDRAB infections is limited, and the new therapeutic regime with combination drug use has been pursued^16,17,20,21^.

Given that biofilms contribute to bacterial virulence and resistance^22^, we targeted agents with anti-biofilm property for their potential in combination drug use against *A. baumannii*. At their sub-MIC levels, our results revealed that the five BFIs tested in this investigation showed different degrees of inhibitory effects on the biofilm formation of XDRAB strains, especially the zinc lactate had the strongest effect, followed by stannous fluoride, furanone, rifampicin and azithromycin at our assay conditions. The inhibition of biofilm formation by these BFIs likely occurs through different mechanisms. Studies have shown that zinc compounds can inhibit the synthesis of extracellular polysaccharides or the formation of matrix networks, and stannous fluoride can destroy the biofilm structure by loosening the structure of the biofilm matrix^13,23^. Furanone, a quorum-sensing system inhibitor, inhibits the biofilms formation of bacterial by replacing the binding sites of quorum sensing signal molecules^24^. Azithromycin can inhibit the synthesis of alginate in the biofilm of *Pseudomonas aeruginosa*, thereby destroying the biofilm structure, leading to the formation of channels on the biofilm that may allow the synergistic drug to pass through the biofilm and thus to reach and kill the bacteria inside^25^. Our findings of the effects of sub-inhibitory zinc lactate, stannous fluoride and furanone further expand the understanding of sub-inhibitory conventional antibiotics including azithromycin and rifampicin in the reduction of *A. baumannii* biofilm formation^26^.

Although BFIs of zinc lactate, stannous fluoride, furanone, and azithromycin have effects on XDRAB, their MIC values are high. Only rifampicin has a low MIC value. However, for the latter, the rapid development of RNA polymerase subunit-encoding *rpoB* gene mutation-mediated resistance to rifamycins limits the use of rifampicin alone against bacterial infections including *A. baumannii*^27^. According to the drug treatment principles for XDRAB, carbapenems (imipenem or meropenem), polymyxins (colistin or polymyxin B) and tigecycline are used as basic drugs in combination with other types of antibacterial drugs. Therefore, there is a clinical value in exploring how these BFIs interplay when an anti-*A. baumannii* antibacterial drug is used in combination with a BFI. The combination drug susceptibility testing results, as shown in Table 2, mainly reveals different levels of synergistic and additive antibacterial effects on XDRAB isolates, which suggests that BFIs may exert their actions via the reduction of biofilm formation and/or direct effect on bacterial growth to interplay with anti-*A. baumannii* antibiotics against XDRAB. With respect to rifampicin or azithromycin combination use with one of 4 anti-*A. baumannii* antibiotics, our results are largely in agreement with published in vitro studies^28–30^. The synergistic or additive effect from the combination use of zinc lactate, stannous fluoride, furanone or rifampicin with imipenem or meropenem against carbapenem-resistant XDRAB is not totally unexpected because the BFIs exerts different modes of action from that of carbapenems. An in vivo study has demonstrated efficacy of imipenem-rifampicin against carbapenem-resistant *A. baumannii*^31^. However, as our studies have limitations that focus on the measurement of in vitro activity, the clinical significance of these observations remains to be determined.

Certain combinations also showed partly indifferent or antagonistic effects. For example, there were antagonistic and indifferent effects occurring in 100% of the isolates when zinc lactate and stannous fluoride were each combined with polymyxin B, respectively. This could partly be explained by that the tested XDRAB isolates were not resistant to polymyxins and anti-*A. baumannii* activities of polymyxin B may mask the role of zinc and stannous compounds or these cationic metal compounds may potentially affect polymyxin’s mode of action in disrupting bacterial membrane integrity^32^. In other word, the positively charged group of polymyxin B can bind to the negatively charged phosphate in the phospholipids of the bacterial cell membrane, leading to the death of the bacteria^32^, while zinc lactate and stannous fluoride are metal cation biofilms inhibitors therefore they may compete with polymyxin B for drug targets, showing antagonistic or indifferent effect^33^. In contrast, the observation of indifferent effects from azithromycin combination with imipenem, meropenem, or tigecycline could be attributable to the weak anti-*A. baumannii* activity of azithromycin. In this regard, one study shows that another macrolide, clarithromycin, exerts antagonistic the effect with a carbapenem on *P. aeruginosa*^34^. However, the additivity between azithromycin and polymyxin is likely due to the membrane disruption of XDRAB by polymyxin B that resulted in improved accumulation of azithromycin into the cells and thus antibacterial activity^32,35^.

Furthermore, it is of importance to consider our in vitro drug combination synergistic results from the pharmacokinetic point of view for their potential clinical implications. Azithromycin and rifampicin are systematically administered antibiotics. While an azithromycin concentration comparable to its high MICs for *A. baumannii* is unlikely achievable in an in vivo situation, a pharmacokinetic-pharmacodynamic analysis of rifampicin has indicated to readily obtain pharmacokinetic parameters (such as the maximum serum concentration [C_max_] value) corresponding to relatively low rifampicin MICs for *A. baumannii*^36^. Thus, our data are in support for the use of rifampicin as one of the combination agents for treatment of XDRAB infections^37,38^. On other hand, the three chemical BFIs, zinc lactate, stannous fluoride and furanone, are not expected to be administered systematically, partly due to toxicity concerns. However, their inhibitory concentrations can be readily reached in topical or local use such that as zinc lactate and stannous fluoride have been used in oral care formulations (e.g., mouthwash with 1.4 mg/ml for zinc lactate or up to 16 mg/ml for stannous fluoride)^39,40^, which warrants further studies in their potential topical use to combat wound infections associated with *A. baumannii*^1,41^.

In conclusion, we have presented data demonstrating the inhibitory effects of five BFIs on the biofilm formation of XDRAB at the sub-inhibitory concentrations and interplay between BFIs and anti-*A. baumannii* antibiotics against XDRAB. Further studies are warranted for their potential clinical implications in combating biofilm-associated bacterial infections.

## Methods

### Bacterial strains

Nine isolates of XDRAB were derived from clinical specimen (septum, endotracheal aspirate or respiratory lavage fluid) of patients from the critical care units, geriatrics, internal medicine and emergency department at the First Affiliated Hospital of Chengdu Medical College in 2018 to 2019 (Chengdu, Sichuan, China). These isolates were identified by standard laboratory methods and ATB New (bioMérieux, France) and also were further verified by PCR of two genes, 16S rRNA (with primers 5′-CATTATCACGGTAATTAGTG-3′ and 5′-AGAGCACTGTGCACTTAAG-3′) and *bla*_OXA-51_ (with primers 5′-TAATGCTTTGATCGGCCTTG-3′ and 5′-TGGATTGCACTTCATCTTGG-3′)^42,43^. *Staphylococcus aureus* ATCC29213 and *Escherichia coli* ATCC25922 used as quality control strains in antimicrobial susceptibility testing were obtained from the American Type Culture Collection (USA).

### Drugs and reagents

A wide range of clinically-used antibacterial agents including ampicillin, ampicillin-sulbactam (2:1), cefepime, ceftazidime, doxycycline, minocycline, amikacin, gentamicin, ciprofloxacin, levofloxacin, chloramphenicol, imipenem, meropenem, tigecycline, polymyxin B, azithromycin and rifampicin were purchased from Meilun Biological (Dalian, Liaoning, China). Zinc lactate, stannous fluoride and furanone were obtained, respectively, from Yuanye Bio-Technology (Shanghai, China), Macklin Biochemical (Shanghai), and Sigma-Aldrich (St. Louis, Missouri, USA). Bacterial culture media, Muller-Hinton broth (MHB), cation-adjusted MH broth (CAMHB), and tryptic soy broth (TSB) medium were purchased from Haibo Biotechnology (Qingdao, Shandong, China).

### Antimicrobial susceptibility testing

The minimal inhibitory concentrations (MICs) of antibacterial agents and BFIs for *A. baumannii* were determined by the broth microdilution method according to the guidelines from the CLSI^44^. The testing was independently carried out at least twice. Briefly, bacterial cells were inoculated in Luria–Bertani agar medium at 37 °C for 16–20 h, and then resuspended in saline (0.9% sodium chloride) to produce 0.5 McFarland turbidity standard, followed by a 20-fold dilution. Antimicrobial solutions were prepared according to the CLSI^44^. Zinc lactate and stannous fluoride were prepared in phosphate buffered solution (PBS). Furanone was initially dissolved in dimethyl sulfoxide (50%) and then diluted in PBS (the presence of dimethyl sulfoxide at the assay conditions [≤ 1.6%] was not found to affect *A. baumannii* growth or their biofilm formation). Together, 180 μl CAMHB, 10 μl of a serially diluted antimicrobial agent, and 10 μl of new prepared bacterial suspension solution were add into 96-well plate. The value of OD_600_ was measured after incubation at 37 °C for 16–20 h. Less than 0.10 is considered as sterile growth or the colored drugs are observed with the naked eye for the determination of MICs^44,45^.

### Sub-inhibitory concentrations of the biofilm inhibitors

Using the 96-well cell culture plate, 170 μl of TSB medium was added to each well, followed by the addition of 10 μl of PBS (control group) or BFI solution (treatment group) and 20 μl of bacterial suspension solution (at OD_600_ value of 0.12). Six duplicates were set for each strain. The value of OD_600_ was measured after incubation at 37 °C every 6 h. When the concentrations of BFIs were used at 1 × MIC, 1/2 MIC, 1/4 MIC, and 1/8 MIC, the growth curves of nine isolates of *A. baumannii* within 24 h were plotted respectively, and the sub-inhibitory concentrations that did not affect the bacterial growth was finally selected as the working concentration for subsequent experiments^46^.

### Bacterial biofilm formation assay under sub-inhibitory concentrations of the biofilm inhibitors

The biofilm formation assay was performed (with six duplicates for each isolate) using 96-well cell culture plate model with crystal violet staining^47^. The assay was repeated independently twice. Briefly, 170 μl of TSB medium and 10 μl of PBS (control group) or BFI solution (treatment group) were added into each well, followed by the inoculation of 20 μl of bacterial suspension (OD_600_ of 0.12). After incubating at 37 °C for 24 h, non-adherent bacterial cells were removed by washing three times with 200 μl of PBS and dried in air. The remaining adherent bacterial cells were stained by adding 200 μl of 0.1% crystal violet. Following incubation of the plates at room temperature for 15 min, the plates were washed three times with PBS and then dried in air. Subsequently, 200 μl of 95% ethanol was added to each well for 5 min and the absorbance of the decolorization solution from each well was measured at the wavelength of 570 nm.

### Combination antimicrobial susceptibility testing

The synergistic antibacterial effects of each of the five BFIs with one of the four anti-*A. baumannii* antibiotics on nine XDRAB isolates were evaluated by microdilution checkerboard method for determining FICI values. The highest concentration of each agent was 2 times its MIC with twofold sequential dilutions, and 8 concentration gradients were tested. Briefly, 170 μl of MHB, 10 μl of each of the series diluents of agents A and B, and 10 μl of freshly prepared bacterial suspension solution were added into the 96-well plate. The checkerboard consisted of columns in which each well contained the same amount of the drug being diluted along the x-axis, and rows in which each well contained the same amount of the drug being diluted on the y-axis, which resulted in each well of a checkerboard containing a unique combination of the two agents being tested. The results were visualized by incubation at 37 °C for 16–20 h. The FICI values were determined for two agents (A and B) by the equation: FICI = A (MIC_combined_ /MIC_alone_) + B (MIC_combined_/MIC_alone_), with the interpretive criteria as: FICI ≤ 0.5 for synergy, 0.5 < FICI ≤ 1 for additivity, 1 < FICI ≤ 2 for indifference, and FICI > 2 for antagonism^48,49^.
